# Reducing stigma among healthcare providers to improve mental health services (RESHAPE): protocol for a pilot cluster randomized controlled trial of a stigma reduction intervention for training primary healthcare workers in Nepal

**DOI:** 10.1186/s40814-018-0234-3

**Published:** 2018-01-24

**Authors:** Brandon A. Kohrt, Mark J. D. Jordans, Elizabeth L. Turner, Kathleen J. Sikkema, Nagendra P. Luitel, Sauharda Rai, Daisy R. Singla, Jagannath Lamichhane, Crick Lund, Vikram Patel

**Affiliations:** 1 Duke Global Health Institute, Duke University, Durham, USA; 2 Transcultural Psychosocial Organization Nepal, Baluwatar, Nepal; 3 Department of Psychiatry, George Washington University, 2120 L St NW, Suite #600, Washington, DC 20037 USA; 4 King’s College London, Centre for Global Mental Health, Institute of Psychiatry, Psychology and Neuroscience, London, UK; 5 Department of Biostatistics and Bioinformatics, Duke University, Durham, USA; 6 Department of Psychology and Neuroscience, Duke University, Durham, USA; 7 Department of Psychiatry, Sinai Health System and University of Toronto, Toronto, ON Canada; 8 Lunenfeld-Tanebaum Research Institute, Toronto, ON Canada; 9 Independent Citizens Coalition Nepal, Kathmandu, Nepal; 10 Alan J Flisher Centre for Public Mental Health, Department of Psychiatry and Mental Health, University of Cape Town, Cape Town, South Africa; 11 Sangath, Porvorim, Goa India; 12 Harvard T.H. Chan School of Public Health, Harvard University, Boston, USA; 13 Department of Global Health and Social Medicine, Harvard Medical School, Boston, USA

**Keywords:** Attitudes, Competence, Low- and middle-income countries, Mental health, Non-specialists, Primary care, Service users, Task-shifting, Training, Stigma

## Abstract

**Background:**

Non-specialist healthcare providers, including primary and community healthcare workers, in low- and middle-income countries can effectively treat mental illness. However, scaling-up mental health services within existing health systems has been limited by barriers such as stigma against people with mental illness. Therefore, interventions are needed to address attitudes and behaviors among non-specialists. Aimed at addressing this gap, *RE*ducing *S*tigma among *H*ealthc*A*re *P*roviders to Improv*E* mental health services (RESHAPE) is an intervention in which social contact with mental health service users is added to training for non-specialist healthcare workers integrating mental health services into primary healthcare.

**Methods:**

This protocol describes a mixed methods pilot and feasibility study in primary care centers in Chitwan, Nepal. The qualitative component will include key informant interviews and focus group discussions. The quantitative component consists of a pilot cluster randomized controlled trial (c-RCT), which will establish parameters for a future effectiveness study of RESHAPE compared to training as usual (TAU). Primary healthcare facilities (the cluster unit, *k* = 34) will be randomized to TAU or RESHAPE. The direct beneficiaries of the intervention are the primary healthcare workers in the facilities (*n* = 150); indirect beneficiaries are their patients (*n* = 100). The TAU condition is existing mental health training and supervision for primary healthcare workers delivered through the Programme for Improving Mental healthcarE (PRIME) implementing the mental health Gap Action Programme (mhGAP). The primary objective is to evaluate acceptability and feasibility through qualitative interviews with primary healthcare workers, trainers, and mental health service users. The secondary objective is to collect quantitative information on health worker outcomes including mental health stigma (Social Distance Scale), clinical knowledge (mhGAP), clinical competency (ENhancing Assessment of Common Therapeutic factors, ENACT), and implicit attitudes (Implicit Association Test, IAT), and patient outcomes including stigma-related barriers to care, daily functioning, and symptoms.

**Discussion:**

The pilot and feasibility study will contribute to refining recommendations for implementation of mhGAP and other mental health services in primary healthcare settings in low-resource health systems. The pilot c-RCT findings will inform an effectiveness trial of RESHAPE to advance the evidence-base for optimal approaches to training and supervision for non-specialist providers.

**Trial registration:**

ClinicalTrials.gov identifier, NCT02793271

## Background

Research trials have shown that non-specialist providers—individuals with no formal training in mental health including primary care and community health workers—can effectively deliver mental health services in low-resource settings in low- and middle-income countries (LMIC) [1, 2]. However, scaling-up these services into government and non-government health systems requires addressing numerous implementation barriers. One limiting factor is the incomplete uptake from training into service provision and lack of fidelity to evidence-based practice (EBP) among non-specialists. This is due, in part, to negative attitudes and other forms of stigma among health workers against persons with mental illness [3, 4].

Attitudes among providers are critical in influencing the adoption of EBP [5–10]. When non-specialist and specialist health workers have negative attitudes toward patients, they are less likely to implement EBP, they spend less time with patients with mental illness, and they allow fewer opportunities for patients and their families to share concerns [11–14]. With similarities to high-income countries (HIC), negative beliefs among healthcare workers in LMIC are widespread including beliefs that people with mental illness are violent, they are to blame for their illnesses, that they can only be treated by specialists, and that treating a person with mental illness can transmit the illness to the healthcare provider [4, 15–19]. Moreover, studies in LMIC have documented that health workers often choose not to provide mental healthcare because of the stigma against mental illness [20–25]. Furthermore, the risk of providers having negative attitudes increases when engagement in new tasks is perceived as involuntary [26]. This may be commonplace in task-sharing (also referred to as task-shifting), where non-specialists perceive obligatory engagement with new responsibilities imposed by government and non-governmental institutions as adding to existing service burdens [5].

Interventions are needed to address attitudes among non-specialist providers to enhance delivery of quality mental healthcare. Improving provider attitudes and competence can increase the likelihood that interventions are scaled up and delivered with fidelity. Lessons learned from the field of HIV/AIDS treatment suggest that reducing healthcare worker stigma can improve care and patient outcomes. Training programs for healthcare workers that integrate stigma-awareness and stigma reduction techniques have been successfully implemented in HIV counseling in sub-Saharan African and other low-resource settings [27, 28]. At a national referral hospital in Tanzania, healthcare workers who participated in a stigma reduction program in addition to HIV/AIDS counseling training had more positive patient outcomes, including increased uptake of antiretroviral treatment among patients as well as higher disclosure rates, compared to healthcare workers who were only trained in HIV counseling [29]. Similar results demonstrating improved patient care (e.g., increased access to HIV prevention programs, increased awareness of and reduced engagement in discriminatory practices in hospitals) through the reduction of health worker stigma have been achieved in settings ranging from Vietnam to the Southeast United States [28, 30, 31].

Unlike trials in the field of HIV/AIDS care, there is a dearth of rigorous anti-stigma trials in the field of mental health in LMIC and a global lack of information on sustained changes in attitudes and clinical competence [4, 15, 32], even though stigma has been demonstrated to be such an important barrier for mental healthcare [33–37]. A review of the literature on stigma research in LMIC identified few intervention studies (*n* = 27), of which only six reported any outcomes and none included long-term follow-up of participants, and the authors concluded that social contact interventions have not been sufficiently evaluated for effectiveness [15].

To address this gap, we propose *RE*ducing *S*tigma among *H*ealthc*A*re *P*roviders to improv*E* mental health services (RESHAPE) as an intervention for non-specialist healthcare providers (specifically, primary healthcare workers) in which social contact with mental health service users is added to training programs. RESHAPE is grounded in the increasing evidence that contact-based interventions can improve attitudes and decrease stigma [4, 32, 38]. Contact interventions include facilitated interaction with mental health service users, such as mental health service users participating in training by giving testimonials and sharing social activities [39, 40] and reflect a broader literature of patient-centered research [41]. Contact interventions perform better than education interventions with in-person contact showing a greater mean effect size for attitudinal change and behavioral intentions compared to video contact; however, among adolescents, only in-person social contact outperforms education, whereas video contact has small mean effect sizes [38].

**Fig. 1:**
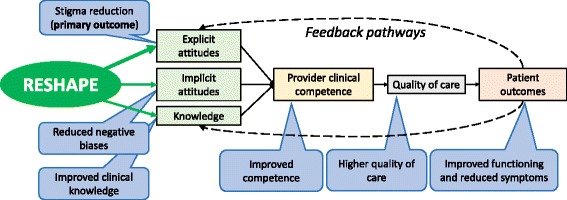
Conceptual model for REducing Stigma among HealthcAre Providers to improvE mental health services (RESHAPE). The RESHAPE intervention utilizes social contact with persons with mental illness who are trained as RESHAPE co-facilitators to reduce explicit stigmatizing attitudes as well as enhance uptake of knowledge, reduce negative implicit biases, and subsequently improve clinical competence, quality of care, and patient outcomes

The RESHAPE implementation strategy is built on this and other evidence from HIV for stigma reduction among health workers including multiple forms of social contact and testimony from trained mental health service users, as well as myth busting, teaching communication skills to address stigma, a recovery emphasis, and use of enthusiastic facilitators [4, 32, 38, 42]. We hypothesize that the RESHAPE interventions reduce stigmatizing attitudes and subsequently leads to better clinical competency, improved quality of care, and optimal patient outcomes through a mechanism of social engagement with service users during training. Engagement with service users, who are trained as RESHAPE facilitators, will lead to greater recognition of patients as human beings worthy of attention and clinical effort, which are components of common factors in mental healthcare that contribute to intervention effectiveness across different modalities of treatment [43–45]. Moreover, the participation of service users fosters beliefs that mental illnesses are treatable and normalize the experience of living with a mental illness. Improved attitudes and enhanced delivery of evidence-based treatments can then be mutually reinforcing [46] leading to improved patient outcomes, and over time, experiences with positive patient outcomes will further reinforce improved knowledge and positive explicit and implicit attitudes (see Fig. 1). Ultimately, RESHAPE is designed as a potential addition to any mental health training for non-specialists to improve provider attitudes and competence as a pathway of improving patient outcomes.

Pilot studies are needed to address issues related to process, resources, management, or scientific approaches [47, 48]. In the case of the RESHAPE trial, we are conducting a pilot for *process* reasons, such as determining recruitment and retention of mental health service users to participate as facilitators and retention of health workers when trained by mental health services users. Additionally, we need to determine the ability of a full trial to capture both direct benefits on health workers and indirect benefits on patients, with the latter necessitating feasible recruitment and retention procedures. The second justification for the pilot study is *scientific* questions with regard to the safety of mental health service users participating as facilitators in RESHAPE trainings and issues related to amount of acceptable participation by service users. An additional benefit in the scientific domain is obtaining context-specific preliminary estimates of the degree of clustering of outcomes by health facility and of possible trajectories of outcomes over time, which is relevant for the design of the full cluster trial.

### Objectives

The purpose of the current protocol is to describe a pilot and feasibility study employing a cluster randomized trial (c-RCT) with two arms incorporating qualitative and quantitative data collection. The two trial arms are training as usual (TAU) and RESHAPE. We will assess the acceptability and feasibility of the RESHAPE intervention, and we will collect data for design of a full-scale effectiveness c-RCT of RESHAPE compared to TAU. The pilot and feasibility objectives (see Table 1) include the following:

1. Establish feasibility and acceptability of involving mental health service users in mental healthcare training and supervision (primary objective);
2. Establish a fidelity evaluation system, and characterize degree of fidelity and degree of contamination;
3. Demonstrate adequacy of randomization procedures;
4. Determine recruitment and retention rates;
5. Establish acceptability, feasibility, and validity of outcome measures;
6. Determine psychometric properties of outcome measures in a clustered design;
7. Demonstrate ethics and safety of the c-RCT protocol;
8. Describe changes in primary care workers’ attitudes, knowledge, and clinical competency;
9. Describe changes in patient-related barriers to care, functioning, and symptoms.

**Table 1:** Pilot study objectives

Domains	Research questions	Hypotheses	Methods	Participants
1-1. Feasibility and acceptability of intervention	Do mental health expert trainers, primary care trainees, and mental health service users find it acceptable for trained mental health service users to participate as co-facilitators in training and supervision?	Key stakeholders will find participation of trained mental health service users acceptable.	● Qualitative interviews with mental health expert trainers, primary care trainees, trained mental health service users, and research staff ● Process notes from trainings and supervision sessions	Mental health expert trainers, primary care trainees, trained mental health service users, research staff
1-2. Fidelity and contamination of intervention	Can fidelity be feasibly and reliably assessed? What degree of fidelity to RESHAPE is achievable? Can contamination be captured through fidelity and other assessments?	Fidelity can be feasibly and reliably assessed with a structured tool, which will also inform assessment of contamination	● Use of fidelity assessment tool by research staff; *target is fidelity of 75% of items on the fidelity checklist* ● Qualitative interviews with mental health expert trainers, primary care trainees, trained mental health service users, and research staff ● Process notes and videos from trainings and supervision sessions	Mental health expert trainers, primary care trainees, trained mental health service users, research staff
1-3. Randomization	Are there biases in the randomization procedure for primary care workers or patients? How could randomization be adjusted based on contamination findings?	Simple randomization will be adequate	● Tabulation of descriptive summaries for baseline characteristics comparing the two groups ● Trainee demographics (educational/professional qualifications, age, gender, prior mental health exposure, years of experience) ● Health facility log book review for patient demographics (age, gender, disorder, number of visits)	Primary care trainees and patients
1-4. Recruitment and retention	Can adequate numbers of mental health service users be recruited, trained, and retained to serve as facilitators? Can adequate numbers of primary care workers and patients be recruited and retained for outcome analyses?	Mental health service users can be trained and retained throughout to sustain ongoing social engagement throughout the study. Primary care workers and patients will need to be over-recruited to account for population mobility, loss to follow-up, and professional transfers.	● Process outputs: mental health service users (number trained, number participating in training, number participating in supervision), *target is 50% service user retention*; primary care worker trainees (number available in facilities, number at trainings, number at supervision sessions; number completing assessments), *target is 66% health workers completion of assessment at 16 months*; patients (number attending facilities, number of sessions received, number consenting, number completing assessments), *target is 66% patient completion of 6-month follow-up assessment* ● Qualitative interviews with primary care trainees, trained mental health service users, patients, and research staff	Primary care trainees, trained mental health service users, patients, research staff
1-5. Acceptability, feasibility, and validity of measures	Are the assessment tools feasible to administer and understand for primary care workers and patients at the planned intervals? Is there expected inter-instrument validity?	The measures will demonstrate adequate acceptability, feasibility, and validity for subsequent trials.	● Tool completion rate, time for completion, number of missing items; *target is fewer than 15% missing items on measures* ● Correlations among instruments ● Cognitive interviewing for transcultural validity	Primary care trainees, patients, and research staff
1-6. Instrument statistical characteristics in cluster design	What is the between and within cluster variance for outcome measures?	Clustering of outcomes within health facilities supports need for cluster randomized design	● Statistical analyses of outcome measures	Primary care trainees, patients
1-7. Ethics and safety of trial	Does the research pose harm to primary care workers, patients, or mental health service users, facilitators and are these harms adequately prevented, documented, and addressed?	A subsequent larger scale trial can be conducted using the ethical and safety standards piloted	● Qualitative interviews ● Process evaluation notes ● Documentation of adverse events and serious adverse events	Primary care trainees, patients, mental health expert trainers, mental health service users, and research staff
1-8. Assess the change in primary care worker attitudes, knowledge, and clinical competency	Do primary care workers’ knowledge, attitudes, and competence improve?	Primary care workers in the RESHAPE intervention arm will show improvement in outcomes	● Outcome assessment pre- and post-training, plus 4- and 16-month follow-up	Primary care trainees
1-9. Assess the change in patient stigma-related barriers to care, functioning, and symptoms	Do patients’ experiences of stigma, functioning, and depression symptoms improve?	Patients in the RESHAPE intervention arm will show improvement in outcomes	● Pre-treatment assessment plus 6-month follow-up	Patients

## Methods/design

### Setting

The study will take place across 34 healthcare facilities (each healthcare facility represents one cluster in the design) in Chitwan district in southern Nepal. Nepal is a low-income country in Asia and is categorized by the World Bank as a fragile state [49]. Nepal’s population is approximately 27 million with the majority (83%) of the population living in the rural areas [50]. The country suffered a decade-long civil war from 1996 to 2006 as well as two major earthquakes in 2015. Previous studies have demonstrated the impact of political violence on psychosocial wellbeing and mental health in Nepal, showing high rates of depression ranging from 17 to 40% since the conclusion of conflict [51, 52]. After the earthquakes, depression was found to affect one out of three adults [53]. In Nepal, depression is also associated with impaired functioning [52, 54], and suicide is the leading cause of death among women of reproductive age in Nepal [55]. One out of 10 adults presenting to primary care services endorses recent suicidal ideation [56]. This is in the context of limited specialized mental health services in both Nepal and throughout South Asia [57]. In 2011, there were fewer than 75 Nepali psychiatrists in clinical practice, with the majority of these working in large urban areas or outside of Nepal in HIC [58].

It is within a context of recent violence and natural disasters, ongoing poverty, high depression burden, and lack of mental health services that the UK AID/DFID-sponsored Programme for Improving Mental healthcarE (PRIME) is being implemented in Chitwan, Nepal, over the period of 2012–2019 [59]. PRIME aims to improve the coverage of treatment for priority mental disorders by implementing and evaluating a comprehensive mental healthcare package, integrated into primary healthcare in five LMICs (Nepal, India, South Africa, Ethiopia, and Uganda) [60–66]. Prior to the implementation of PRIME, no mental health services were systematically available in primary healthcare in Nepal [58, 62].

Government health facilities include health posts, primary health centers, urban clinics, and hospitals. These are all part of the government health center and represent the first portal for care. In these facilities, primary healthcare workers include health assistants, community medical assistants, and auxiliary nurse midwives, all of whom are non-specialist with approximate 2 years of medical training. Some health facilities also include medical doctors with MBBS (bachelor of medicine/bachelor of surgery) credentials. PRIME and the RESHAPE component are implemented by Transcultural Psychosocial Organization (TPO) Nepal, a Nepali non-governmental mental health research and training organization [67]. The PRIME program is divided into two phases: implementation and scale-up. During the implementation phase, the PRIME package of care was piloted in 12 health facilities in Chitwan [59]. In the scale-up phase, the PRIME program is expanded to the remaining 34 health facilities in Chitwan district. The RESHAPE vs. TAU study will take place in all 34 health facilities participating in the scale-up phase. Additional information on the study site in Chitwan has been previously published [59].

### Design

We will compare TAU to the novel training strategy (RESHAPE). We will employ a c-RCT in which primary care health facilities in Chitwan are randomly assigned to either the TAU or RESHAPE. A healthcare facility cluster design was selected because attitudes and clinical behaviors are influenced by peers, and thus, we anticipate a high degree of contamination among primary healthcare workers within a facility. Moreover, clinical care is not restricted to exclusive relationships with a single provider in the government health system. Therefore, patient care will be characterized by seeing a range of healthcare workers within a single facility over the course of their treatments. Therefore, a cluster design with the health facility as the unit of clustering is required to minimize contamination.

### Intervention: training as usual versus training augmented with the RESHAPE intervention

The TAU arm will include the standard PRIME training based on the district mental health plan developed for PRIME in Nepal [59]. There are two versions of the standard PRIME training: one for prescribers and one for non-prescribers, a division based on scope of practice under government care regulations. Prescribers refer to health workers who can prescribe medication (health assistants and auxiliary health workers). We have selected these paramedical staff rather than focusing exclusively on MBBS-credentialled doctors because not all primary healthcare facilities have doctors on staff. Therefore, health assistants and auxiliary health workers are most often the front-line of primary healthcare. MBBS doctors are limited to a few primary healthcare centers and most hospitals and are usually seen after a person has encountered a lower level cadre of paramedical workers.

Training for prescribers in PRIME is based on the World Health Organization (WHO) mental health Gap Action Programme (mhGAP)-Intervention Guide [68] and includes a range of neuropsychiatric disorders, of which four were selected for the focus of PRIME in Nepal: depression, psychosis, epilepsy, and alcohol use disorder. Psychosocial modules focusing on communication skills, supportive techniques, and health education are included based on prior curricula and adapted for Nepal [69]. Training to prescribers is provided over 10 days and delivered by a psychiatrist and an experienced psychosocial counselor. Following training, the prescriber group participates in supervision sessions with a psychiatrist, with supervision conducted approximately once every 3 months.

In the Nepal health system, non-prescribers cannot prescribe medication and provide community outreach, assist in vaccination within maternal and child health programs, and promote treatment adherence. Non-prescribers are predominantly auxiliary nurse midwives. Within PRIME, non-prescribers deliver psychological treatments including general psychosocial care, the Healthy Activity Program (HAP) intervention, which is a six to eight session behavioral activation psychological treatment that enhances participants’ uptake of pleasurable and mastery activities with the objective of depression symptom reduction, and the Counseling for Alcohol Problems (CAP) intervention, which is a two to four sessions simplified and adapted version of motivational interviewing for treatment of alcohol use disorder [70]. Both HAP and CAP have demonstrated effectiveness for depression and harmful alcohol use respectively when delivered by non-specialist providers in India [71, 72]. Non-prescribers receive 5 days of training in psychosocial fundamentals and then a select group receives an additional 5 days of training in HAP and CAP. Non-prescribers receive individual on-the-job supervision from experienced psychosocial counselors.

RESHAPE uses the basic model of PRIME training and supervision. For both the prescriber (10-day) and non-prescriber (5-day basic psychosocial plus 5-day HAP/CAP) training, mental health service users will participate as co-facilitators. Patients with mental illness who are in recovery after receiving treatment through the PRIME original implementation areas will be trained to serve as mental health service user co-facilitators in the RESHAPE program in the scale-up health facilities. Mental health service users are trained to become co-facilitators using “PhotoVoice”—a participatory research approach in which photography is used to develop testimonials and other messaging [73–76]. PhotoVoice has previously been used with illiterate women with depression in rural Nepal [77]. Service user co-facilitators in RESHAPE provide personal testimonials, ongoing social contact, and myth busting and exemplify a recovery emphasis [42]. In addition, a health worker who has previously participated in the PRIME program (in the original implementation health facilities) receives training to participate as a co-facilitator and serves as an enthusiastic and aspirational role model [42]. The rationale for the aspirational figure is to provide a linkage between the current identity of the primary care trainees and the role they would like to achieve by the end of the training. Table 2 describes the key elements of the RESHAPE intervention.

**Table 2:** Elements of RESHAPE intervention

RESHAPE elements	Description of element content	Implementation of element
Engagement with service users	Opportunities for socialization, participation in practice role plays, collaborative problem solving	Included during multiple days of training
Testimonial from service users	Three-part testimonials developed through PhotoVoice training using photographs and personal stories to describe life before treatment, the experience of treatment, and life after treatment	Testimonials provided separately for target disorders: depression, psychosis, alcohol use disorder, and epilepsy
Testimonials from aspirational figures	Three-part testimonials describing experiences and attitudes prior to mental health training, experiences of providing mental healthcare, and changes in attitude and behavior after starting delivery of mental health services	One or two testimonials from health workers who previously participated in PRIME training and mental health service delivery
Myth busting	Eight common myths: mental illness cannot be treated; only some people can get mental illness; mental illnesses are contagious; mental illness can only be treated with shots and pills; giving advice is the same thing as doing psychological counseling; all people with mental illness are violent; if you ask someone about suicide, that increases the risk they will kill him/herself; caring for people with mental illness makes you mentally ill	Delivered in one session by one aspirational primary care worker
Didactic session on stigma and discrimination	Definitions of stigma and discrimination; reasons for stigma and discrimination; addressing different causes of discrimination: peril stigma, occupational stigma, and social stigma	Delivered in one session by a trained facilitator working for the PRIME implementation NGO (TPO Nepal)

Patients in both the TAU and RESHAPE arms receive the same intervention package (mhGAP plus psychosocial services, HAP, and CAP). No elements of the treatment will vary based on intervention arms. If patients require greater levels of care than those provided through the mhGAP-trained primary healthcare workers, patients in either arm can be referred to psychiatrists at the local psychiatric specialty service for any concerns related to diagnosis, medication management, or psychiatric emergencies. Patients are not discontinued from either treatment arm if referral is required and can continue to participate in follow-up evaluations. Patients are included in the current pilot study because we plan to test whether the RESHAPE training paradigm leads to improved patient outcomes in the subsequent full-scale effectiveness trial. Specifically, in the subsequent full trial, we will evaluate if RESHAPE-trained primary healthcare workers have greater clinical competency and deliver mhGAP, HAP, and CAP with greater quality resulting in better patient functioning and greater symptom reduction in depression (HAP participants) and harmful drinking (CAP participants).

### Participants

**Fig. 2:**
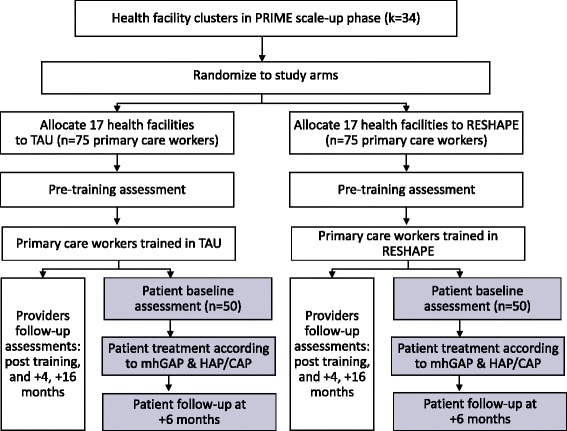
Flow chart for RESHAPE pilot cluster randomized controlled trial. Flow diagram for progress of health facility clusters and primary healthcare workers (PCW). Gray boxes represent patient flow. Abbreviations: PRIME Programme for Improving Mental healthcarE, RESHAPE Reducing Stigma among HealthcAre Providers to ImprovE mental health services, mhGAP mental health Global Action Programme, HAP Healthy Activity Programme, CAP Counseling for Alcohol Problems

For the pilot c-RCT, we plan to recruit all primary healthcare workers (approximately 150 including prescribers and non-prescribers) who are participating in the PRIME scale-up phase health facilities (*k* = 34 facilities), and a subset of their patients, approximately 100 (see Fig. 2). The health facilities will include 32 primary care centers and 2 hospitals providing primary care services. Primary healthcare workers are the intended direct beneficiaries of RESHAPE. Their patients are the intended indirect beneficiaries. In the pilot, additional participants will include mental health service users who will be trained as facilitators for RESHAPE, mental health experts (psychiatrists and psychosocial counselors) who serve as facilitators and supervisors, and research staff (research assistants, field coordinators, and external competency and fidelity raters).

### Inclusion criteria

All primary healthcare workers (approximately 150) participating in either the prescriber (10-day mhGAP) or non-prescriber (5-day basic psychosocial with some non-prescribers also participating in the 5-day additional HAP/CAP training) PRIME trainings will be invited to participate. We anticipate the majority of primary healthcare workers will be paramedical staff. However, if MBBS doctors are working at primary healthcare centers and included in the training, they will also participate in the RESHAPE pilot trial. Any patient receiving PRIME services will be invited to participate. This includes persons with diagnoses of depression, psychosis, alcohol use disorder, or epilepsy made by prescribers using mhGAP guidelines.

### Exclusion criteria

Primary healthcare workers who do not have appropriate government credentialing will be excluded. Patients who are personally unable to complete the research interview as well as those lacking caregivers able to assist with the assessments will be excluded.

### Measures/outcomes for feasibility criteria and other objectives

The primary objective is to evaluate feasibility and acceptability (see the “Objectives” section). In accordance with recommendations for pilot study reporting [48], we have established qualitative and quantitative indicators to guide decisions about what procedures to carry through to the full trial and when modifications should be made. Acceptability and feasibility will be evaluated through qualitative interviews and focus group discussions with primary care health workers. Other qualitative topics will be acceptability and feasibility of taking on mental health services. Adoption of mental health services will be measured as the amount of mental health services (e.g., number of outpatient visits) provided in the primary care facilities. The other objectives will be to evaluate fidelity, randomization, recruitment and retention, acceptability and feasibility of measures, instrument statistical characteristics, and ethics and safe conduct of the trial. Taking these objectives together, overall feasibility and acceptability will be determined by the following criteria to determine progression to the full trial:

- Identification of qualitative themes reporting that service user co-facilitation benefits acquisition and implementation of knowledge, instills positive attitudes toward persons with mental illness, e.g., “see the person” messages, messages that “mental illnesses are treatable”, and “recovery messages,” [78] and the absence of themes that service users are perceived as disruptive. Among service users, the qualitative data will be coded for themes that participation is empowering or enhancing self-efficacy, that participation does not damage familial or community relations, that participation is perceived as safe, and that participation is not perceived as stressful resulting in worsening mental health (domain 1-1)
- Fidelity to 75% of items on the fidelity checklist for RESHAPE elements (domain 1-2)
- Tabulation of descriptive summaries for baseline characteristics comparing two groups of primary care workers demonstrating no significant group differences in educational/professional qualifications, age, gender, primary mental health exposure, and years of experience (domain 1-3)
- Retention of at least 50% of mental health service users as facilitators in the RESHAPE trainings, 66% completion of 16-month follow-up by health workers, and 66% follow-up of patients (domain 1-4)
- Fewer than 15% missing items on outcome measures (domain 1-5)
- Presence of adverse events among fewer than 10% of participants and any serious adverse events (domain 1-7)

In domains where criteria are met, we will retain the procedure for the full trial. In domains where criteria are not met, we will modify procedures for the full trial. The presence of any adverse events and serious adverse events will be addressed by the trial team to identify alternative strategies for the full trial.

In addition, there are two objectives related to gathering pilot quantitative data for both primary healthcare workers’ and patients’ outcomes (domains 1-8 and 1-9 in Table 1). For these outcomes, we will complete descriptive reports of reduction in mental illness stigma among primary healthcare workers operationalized as a reduction in social distance scales, which is the intended primary outcome of the subsequent full trial. The primary outcome for the eventual full study is the Social Distance Scale (SDS) previously used in another study in Nepal and based on select sections of the instrument used in the Stigma in Global Context—Mental Health Study [79–81]. The Nepali version of the SDS for healthcare workers has 12 items and usage in PRIME prior to the RESHAPE study found that the SDS has strong internal consistency (*α* = 0.80). Secondary health worker outcomes for the full trial include knowledge, implicit biases, and clinical competency (see Table 3). There will also be a range of secondary outcomes for primary healthcare workers. A secondary measure of stigma will be the mhGAP attitude battery previously used in PRIME in Nepal and other global PRIME sites. Similarly, the PRIME mhGAP knowledge assessment (multiple choice and true-false questions) will be used. Implicit biases will be assessed with a computer-based implicit association test (IAT) [82] adapted for use with stimuli appropriate for Nepali health workers [83]. Competence will be assessed through standardized role plays with mock patients. The role plays will be scored with the ENhancing Assessment of Common Therapeutic factors (ENACT) rating scale. ENACT was developed in Nepal within PRIME [84, 85]. In addition, psychiatrists will interview a subset of patients diagnosed by primary healthcare workers. The psychiatrists will use the Composite International Diagnostic Interview (CIDI), which has been validated in Nepal [86], to establish a diagnosis and then compare with the primary healthcare worker’s diagnosis and treatment recommendations as a measure of treatment fidelity. We will also attempt to contact primary healthcare workers who drop out of the study (e.g., get reassigned to another health facility, retire, change profession) for quantitative and qualitative interviews to assess impact of TAU vs. RESHAPE on subsequent professional activities.

**Table 3:** Pilot c-RCT outcome measures

Construct	Instrument	Description	Assessment time periods					
			Pre-training (*t*_1_)	Immediate post-training (*t*_2_)	4-month post-training (*t*_3_)	16-month post-training (*t*_4_)	Patient baseline (*t*_5_)	Patient 6-month follow-up (*t*_6_)
Primary outcome (primary healthcare workers)								
Stigma	Social distance	Primary healthcare workers self-rate level of social distance related to interacting with persons with mental illness	X	X	X	X		
Secondary outcomes (primary healthcare workers)								
Mental healthcare knowledge	mhGAP knowledge	Primary healthcare workers complete multiple choice and true/false questions reflecting knowledge of mental health diagnoses and treatment	X	X	X	X		
Stigma	mhGAP attitudes	Primary healthcare workers complete questions regarding attitudes toward people with mental illness	X	X	X	X		
Stigma	Implicit Association Test (IAT)	Primary healthcare workers complete a computer-based neuropsychological test assessing implicit biases related to mental illness and violence	X		X	X		
Clinical competency	Enhancing Assessment of Common Therapeutic Factors (ENACT)	Competency is rated by observers through role plays between primary healthcare workers and standardized patients	X		X	X		
Diagnostic and treatment fidelity	Psychiatrist administered Composite International Diagnostic Interview (CIDI)	Psychiatrists administer the CIDI to patients diagnosed by primary healthcare workers and compare with the diagnosis and treatment recommendations					X	
Secondary outcomes (patients)								
Stigma and care access	Barriers to Access to Care Evaluation (BACE)	Patients rate degree to which stigma is a barrier to care seeking					X	X
Perceived clinical competency	Enhancing Assessment of Common Therapeutic Factors (ENACT)—patient rating version	Patients rate their primary healthcare workers on quality of common factors in care					X	X
Daily functioning	WHO Disability Assessment Scale (WHODAS)	Patients rate ability to perform daily functioning					X	X
Depression symptoms	Patient Health Questionnaire (PHQ-9)	Patient rate depression symptoms over past two weeks					X	X
Alcohol use disorder	Alcohol Use Disorders Identification Test (AUDIT)	Patients rate alcohol use and associated behavior, as well as daily ethanol consumption					X	X

Outcomes for patients include stigma-related barriers to accessing care, daily functioning, and symptoms. These are descriptive analyses to inform potential effectiveness which will be evaluated in the eventual full trial. Patients will be evaluated after completion of the primary healthcare worker's initial consultation. This will allow time for the primary healthcare workers to develop their treatment skills and ideally for the skills to stabilize. For patient measures, the Barriers to Access to Care Evaluation (BACE) [87] will be used to evaluate the severity of stigma associated with seeking care. BACE is used currently for PRIME in Nepal. The WHO Disability Assessment Scale (WHODAS) [88] has been used previously in Nepal [54, 89, 90], with excellent internal consistency between items (*α* = 0.90) and strong validity with multiple mental health measures for depression (*r* = 0.70, *p* < 0.001), anxiety (*r* = 0.64, *p* < 0.001), and PTSD (*r* = 0.37, *p* < 0.001). The Patient Health Questionnaire (PHQ-9) [91] has been transculturally translated and clinically validated in Nepal [92]. Harmful alcohol drinking will also be assessed with the AUDIT [93], which has previously been used in Nepal [94, 95], as well as daily ethanol consumption. Patients will also complete a patient-rating version of the ENACT to evaluate the primary healthcare worker’s competency in common clinical factors. Patient data collection will be completed using Open Data Kit [96] on Android tablets. Patients who discontinue care will be followed up at the end of the study for both quantitative and qualitative assessments.

This is an external pilot, and therefore, we will not carry quantitative data from the health worker and patient outcomes into the full trial. In accord with recommendations for a priori determination of carrying data forward from pilots to full trials [97, 98], we have determined that contextual and implementation preclude a carry-forward design. The justification for this is that the current pilot is embedded with the larger PRIME initiative in Chitwan, Nepal [59], which is likely to influence outcomes beyond the focus on RESHAPE training models. Because PRIME has gone through iterative development in Chitwan, different approaches to training, supervision, and evaluation have been used. The full trial will be conducted in naïve sites that have not gone through the PRIME development process.

### Randomization

There are 34 health facilities (clusters) in the PRIME scale-up regions of Chitwan. Health facilities will be the selected unit of randomization because they generally function independently from one another. We will examine baseline differences in patients. If there are baseline differences, we will adjust for this variable (e.g., distance from clinic) in the analytical approach. The allocation ratio for health facilities is 1:1. All health workers attending the trainings will be enrolled by TPO research staff. Randomization of health clusters will be performed by the study statistician (ELT) using a random number generator in Stata version 14 software [99]. Health facilities will be notified regarding the dates for their training, but they will be blinded to whether the training is a PRIME or RESHAPE training. Potential sources of contamination across arms are the movement of health workers from a facility in one to a facility in another arm and the movement of patients from residence in one arm’s catchment to a residence in another catchment. These sources of potential contamination will be monitored and addressed in the design of the subsequent full-scale effectiveness trial after completion of this pilot.

### Recruitment

All primary care trainees in PRIME are expected to participate in pre- and post-training plus follow-up evaluations as a condition of their training participation, which is coordinated with the local government through the district public health office. A subset of trainees will be randomly selected to participate in the qualitative component of the study, and this participation is elective. All patient participation is voluntary, and recruitment takes place after patients receive any mhGAP diagnosis from a primary care provider in the study.

### Blinding and concealment

Primary healthcare workers will be blinded to the condition of training. They will not be informed ahead of time that the main variation in trainings is the presence of mental health service users as co-facilitators, as this may bias their responses. Research assistants will be blinded to the training arm, and psychosocial counselors who perform ENACT evaluations will be blinded to the arm. Raters who evaluate the ENACT recordings will be blinded to both treatment arm and to which period of the training and supervision the evaluation is from. No a priori unblinding specifications are established for primary healthcare workers given that the two implementation arms are not associated with different levels of risk. Unblinding is not relevant for patients given that they will be receiving the same intervention. Study statisticians will be blinded to treatment arm during analyses.

### Sample size

The 34 health facilities are staffed by approximately 80 prescribers and 70 non-prescribers. The sample is selected based on feasibility with the PRIME scale-up region of Chitwan. Sample size was not determined based on inference testing given the pilot design of this study [100]. Approximately 100 patients will be enrolled in the patient outcome component. Patient sample size was based on calculations performed for a quality of care study previously planned for PRIME scale-up. Specifically, the target number of patients is 86, for which we will recruit and enroll 100 participants, assuming limited attrition. With a sample of *n* = 86, in which 80% of them receive appropriate diagnosis and initial treatment, the 95% CI will be 70.2 to 88.0%; allowing for some over-sampling, we are aiming for *n* = 100. The sample size is based on precision calculation and assumes a simple random sample from all diagnosed patients.

### Financial incentives

As per government regulations, primary healthcare workers are paid approximately US$16 per day to attend trainings. In addition, they receive transportation, food, and lodging funds. PRIME will cover these costs for health workers. Similar payments are made for the supervision sessions. Patients are not financially compensated but they are provided with transportation cost, food and lodging when required. Health workers and patients are provided with some form of non-financial compensation (e.g., household gift items) if they are participating in the qualitative interviews outside the training days.

### Data management and monitoring

All investigators on the study will have access to primary data. TPO Nepal research supervisors will do quality assurance checks on data collected by research assistants. Data will be stored in both offline and cloud repositories in compliance with data security recommendations of the institutional review boards involved in the study. Storage platforms will include HIPAA-compliant REDCAP and Box. A Data and Safety Monitoring Board (DSMB) has been established in Nepal for oversight of TPO trials including PRIME and RESHAPE. DSMB members include psychiatrists, legal experts, non-governmental organization experts in psychosocial programs, and mental health advocates. No DSMB board members are study staff of PRIME or RESHAPE. Given that this is a pilot c-RCT that is not powered for inference testing, interim statistical analyses with associated stopping guidelines are not planned.

### Planned analyses

#### Qualitative analyses

Focus group discussions (FGDs), key informant interviews, and process evaluation notes will be coded in NVIVO [101] and analyzed using content analysis [102] for themes of cultural acceptability, experience of consumers as trainers, relevance to clinical care, training duration, structure of training, content of training, and follow-up engagement, following approaches used in similar global mental health studies [103]. Coding will be done by multiple independent raters, and inter-rater reliability will be calculated using Kappa scores. Data analysis will be conducted throughout each step to facilitate iterative revision then finalization of the manual. Following the Consolidated Criteria for Reporting Qualitative Studies (COREQ), we will document the process according to the 32-item checklist [104]. Broadly, for domain 1 “research team and reflexivity,” the qualitative research team will include the PI, TPO staff, and research trainees; the degrees will range from MD and PhD to MA and Bachelors; the occupations will include academic medical faculty, NGO staff, and students; there will be both male and female qualitative staff, and staff experience in qualitative research will range from 1 month to greater than 10 years; the relationship with participants will not precede the study; participants will know that research staff are employed by or associated with TPO Nepal, and interviewer characteristics (age, education, region of origin, etc.) will be reported. For domain 2 “study design,” content analysis will be used; selection will be reported as described above; setting features including location and presence of non-participants will be reported; an interview guide will be used; there will be repeat interviews at different times in the training and supervision timelines; audio will be recorded; duration will be documented; data saturation or lack thereof will be reported; and transcripts will not be returned to participants for analysis. For domain 3, there will be approximately four to six coders; the coding tree will be published; themes will be identified in advance with the option to generate additional themes; participants will not provide feedback on the coding; quotations will be presented; data and findings will be consistent; and major and minor themes will be clearly presented.

#### Statistical analyses

The quantitative outcomes of interest will be summarized descriptively using appropriate summary statistics (mean and standard deviation for continuous outcomes and numbers and proportions for categorical outcomes) and graphically over time for the primary healthcare workers (four time points: pre- and post-training, 4 months-, and 16 months-post-training) and patients (two time points: treatment entry and 6 months later) for both study arms. Provider and patient trends over time for each score will be plotted to examine between- and within-person differences and to determine the plausible pattern (e.g., linearity) of those trends. The profiles will be grouped by cluster (the health facility) and by study arm to examine between-cluster and between-arm variation in provider and patient outcomes. Using the first measurements for provider (i.e., prior to beginning the mhGAP training) and patient (i.e., after the first diagnostic visit with an mhGAP-trained primary care provider), preliminary estimates of within- and between-cluster variances, within- and between-primary care worker variances and the intra-class correlation coefficient (ICC) of patient outcomes will be estimated. Such estimates are essential for sample size calculations for the planned full-scale c-RCT design and are often guessed or obtained from other studies, whereas we will obtain context and design-specific estimates using our pilot data [105, 106]. Nevertheless, as noted by Eldridge et al. [105], there are concerns that sample size estimates based on these pilot data could be too small. Because we will collect individual-level data from 34 clusters, our pilot c-RCT size should mitigate some of these concerns. Nevertheless, we plan to power the full trial based on more conservative estimates of the parameters of interest than those obtained from the pilot c-RCT by using the upper bound of the 95% CI for the ICC and by comparing our estimates to those from other studies of similar outcomes to be sure we will increase our estimates if we find them to be considerably smaller than those from other studies. By using such a “triangulation” approach and by obtaining context-specific data, we are confident that we will be able to better design the full-scale c-RCT than in the absence of the pilot c-RCT data. The pilot data will also be used to inform the choice of effect estimate (e.g., difference in slopes or in means at a specific follow-up time point) in the future c-RCT that will build on the current pilot study. Preliminary indicative estimates of differences in primary and secondary outcomes by arm will be obtained. In practice, we will power the future c-RCT predominantly based on magnitudes of effect that are of public health relevance rather than using magnitudes of effects obtained from the pilot study, which will not necessarily be indicative of what could be attained in an appropriately powered larger c-RCT.

#### Mixed methods framework

This pilot study will follow the Good Reporting of A Mixed Methods Study (GRAMMS) guidelines [107]: First, mixed methods are being used to evaluate feasibility and acceptability qualitatively while quantitative information will be used for the design of the full trial. Second, qualitative and quantitative will be assessed generally during the same intervals of the study. Focus group discussions will be conducted pre- and post-trainings around the timing of pre- and post-quantitative assessments. Similarly, follow-up qualitative and quantitative assessments will occur after a few months of practice and 1 year later. Third, both methods will be clearly documented in publications with regard to sampling, data collection, and analysis. Fourth, integration will occur in regard to health workers qualitative descriptions of their stigma, knowledge, and competency, which will be integrated with quantitative scores on these three variables. Fifth, because this is a pilot study, inference testing on the quantitative data are limited; therefore, we cannot compare qualitative and quantitative data with regard to effectiveness of the RESHAPE program. Sixth, insights resulting specifically from integration of qualitative and quantitative findings will be highlighted.

### Ethics and research governance

#### Consent

Consent follows current models within PRIME wherein health supervisors nominate primary healthcare workers for the training. In this case, we are following the ethic of beneficence in that primary healthcare workers are required to attend the training (whichever variant) because to not attend the training would deny their patients access to mental healthcare. However, all primary healthcare workers will be given the opportunity to refuse participation in the research process and follow-up while still participating in training. This is in accord with approaches others have taken in c-RCTs to assure that patients are not denied care: “if a health care professional chooses not to participate in a study, they are in effect denying their patients the potential benefits of participation. Healthcare providers ought to do the best for their patients,” [108]. For patients, we will follow a similar model in which all patients are consented for participation in the data collection process and follow-up, and participation in treatment is not contingent upon research participation. This is because these health facilities currently lack professionals with mental health training. Therefore, to give the patient the option of not participating with a primary care worker trained through PRIME equates to denying care, i.e., current treatment as usual is no treatment at all. Therefore, at a minimum, all patients presenting to clinics within the clusters will have access to basic mental healthcare through PRIME-trained primary healthcare workers regardless of consent or refusal to participate in the data collection process. Any codes linking participant information to personal identifiers or personal health information will be restricted to the TPO Nepal research supervisor. Only de-identified data will be used for analyses.

#### Harms

The main risk factor is psychological distress among mental health service users trained to co-facilitate trainings. If mental health service users acting as co-facilitators do not feel that the training is a comfortable environment, we have outlined contingency plans regarding use of videotaped testimonials and provision of stand-by counselor to provide immediate care to the mental health service users when required. Given a prior training with healthcare consumers in Nepal, we anticipate that consumers will find the training experience a non-distressing experience. Minimal risk of harm from the treatment is anticipated. In addition to these risks with greatest likelihood, another issue to consider is the negative consequences if confidentiality of information obtained in the study (including subject identity as a research participant or information collected during assessments) were compromised.

Treatment may include psychotherapy and/or medication management. All patients are expected to be receiving optimal clinical care at the clinical judgment of primary healthcare workers. Primary healthcare workers are supervised by psychiatrists in Chitwan who can provide information on management and receive referrals for patients with worsening symptoms or other clinical concerns.

All changes in treatment resulting from adverse events or serious adverse events will be reported to the DSMB in Nepal. TPO Nepal is responsible for data collection and storage and making data available to the DSMB, funders, and IRBs for audits when appropriate.

#### Post-trial care

Primary healthcare workers will remain in the region to continue care after the trial pending any transfers by the government. The government has agreed to update the essential medication list so that patients can get access to medications after the trial. Currently, medications are purchased by PRIME and the specific medication and brands are dictated by the the updated essential medication list and supply preferences by the government.

### Dissemination

Findings from the pilot study will be published in academic journals, disseminated through the PRIME network, reported to funders of PRIME (United Kingdom Agency for International Development, UKAID) and RESHAPE (United States National Institute of Mental Health, NIMH). Authorship eligibility will comply with guidelines of the International Committee of Medical Journal Editors, with additional attention to recommendations for equitable representation of researchers from LMIC for academic authorship [109]. The materials for training and implementation of RESHAPE will be made available through the Mental Health Innovation Network (www.mhinnovation.net). In keeping with NIMH recommendations, data will be made publicly available after publication of primary analyses.

### Timescale

Primary healthcare workers will be followed for 2 years from 2016 to 2018 to evaluate retention, changes in knowledge, attitudes, and clinical competence. Patients will be enrolled approximately 18 months after primary healthcare workers are trained. They will be followed-up 6 months after initial enrollment evaluation. Data collection will be completed by the end of June 2018. See Table 4 for schedule of enrollment, interventions, and assessments.

**Table 4:** Schedule of enrollment, interventions, and assessments for RESHAPE

	Study period							
Primary healthcare workers (*direct beneficiaries*)—trained to deliver mental health services								
	Cluster allocation	Enrollment	Post-allocation					Close-out
Time point	*-t*_*1*_	*t*_*0*_	*t*_*1*_	*t*_*2*_	*t*_*3*_	*t*_*4*_	*t*_*5*_	*t*_*6*_
Enrollment								
Allocation	X							
Eligibility screen		X						
Informed consent		X						
Interventions								
PRIME training and supervision (TAU)			←−−−−−−−−−−−−−−−−−−−−−−−−−−→					
RESHAPE training and supervision			←−−−−−−−−−−−−−−−−−−−−−−−−−−→					
Assessments								
mhGAP knowledge			X	X	X	X		
mhGAP attitudes			X	X	X	X		
Social Distance			X	X	X	X		
Implicit Assoc. Test			X	X	X	X		
Health worker—ENACT			X	X	X	X		
Qualitative interviews			X	X	X			X
Patients (*indirect beneficiaries*)—patients treated by TAU or RESHAPE-trained primary healthcare workers								
	Cluster Allocation		Post-allocation					Close-out
Time point**	*-t*_*1*_		*t*_*1*_	*t*_*2*_	*t*_*3*_	*t*_*4*_	*t*_*5*_	*t*_*6*_
Enrollment								
Allocation	X							
Eligibility screen						X		
Informed consent						X		
Interventions								
mhGAP + HAP/CAP							← − →	
Assessments								
Patient—BACE							X	X
Patient—WHODAS							X	X
Patient—PHQ-9							X	X
Patient—AUDIT							X	X
Patient—ENACT							X	X
Qualitative interviews								X

Note: All health facility clusters are allocated are -*t*_1_. Primary healthcare workers are assigned to Training As Usual (TAU) or RESHAPE trainings based on the health facility in which they work. Primary healthcare workers are enrolled and consents at *t*_0_. Primary healthcare workers are administered assessment batteries immediately prior to training at *t*_1_. They then participate in training and subsequent supervision for the duration of the research study. There is an immediate post-training assessment at *t*_2_, followed by a 4-month (*t*_3_) and 16-month (*t*_5_) assessment. Close-out qualitative interviews are conducted with a subset of primary healthcare workers at *t*_6_. Patients are enrolled with TAU or RESHAPE-trained primary healthcare workers according to the allocation of their local healthcare facility. They all receive mhGAP and HAP/CAP interventions. Patient enrollment occurs at approximately 18 months after primary healthcare workers are trained. Patients are assessed at treatment initiation (*t*_5_) and 6 months later at study close-out (*t*_6_). Close-out qualitative interviews are conducted with a subset of patients at *t*_6_

## Discussion

The results of the pilot trial will be used to determine if we can move forward with the same procedures for the full trial in another region of Nepal. If there are qualitative or quantitative indicators of problems with feasibility and acceptability impacting recruitment, retention, randomization, fidelity, or safety, those relevant procedures will be modified. This is an external pilot study, and therefore, data will not be carried forward from this pilot to the full trial. If significant modifications are needed, we will consider the need for an internal pilot in the context of the full trial [97].

There is growing evidence that improving provider attitudes and competence through adding social contact with mental health service users throughout training and supervision increases the likelihood that mental health interventions are scaled up and delivered with fidelity. If shown to be effective, the implications of our results are not limited to Nepal but also have relevance for improving use and quality of EBP for global mental health in LMIC and high-income settings because adopting EBP and enhancing provider motivation are global challenges [110–112]. Developing implementation strategies to reduce stigma is consistent with objectives of the WHO Action Plan 2013–2020 [113] and a goal of the Grand Challenges in Global Mental Health [114].

### Trial status

The trial is open and recruiting. The protocol was last verified on 24 July 2017. Subsequent protocol modifications will be reported to funders, IRBs, and registered with ClinicalTrials.gov. Modifications made since original submission of the grant proposal to NIMH were instituting a post-training follow-up at 4 months because lack of medication release by the government precluded initiation of services. With the use of the 4-month follow-up, the 6-month follow-up was removed because it was too close in time to the 4-month assessment. In addition, because of the delay in initiation of services, the final quantitative follow-up of healthcare workers was modified to 16 months, to coincide with approximately 1 year of service delivery.

## Abbreviations

AUDIT: Alcohol Use Disorder Identification Test
BACE: Barriers to Access to Care Evaluation
CAP: Counseling for Alcohol Problems
c-RCT: Cluster randomized controlled trial
EBP: Evidence-based practices
ENACT: ENhancing Assessment of Common Therapeutic factors
HAP: Healthy Activity Program
HIC: High-income countries
IAT: Implicit association test
LMIC: Low- and middle-income countries
mhGAP: Mental health Gap Action Programme
NIMH: National Institute of Mental Health
PHQ: Patient Health Questionnaire
PRIME: PRogramme for Improving Mental healthcarE
RESHAPE: REducing Stigma among HealthcAre Providers to improvE mental health services
SGC-MHS: Stigma in Global Context-Mental Health Survey
TAU: Training as usual
TPO: Transcultural Psychosocial Organization Nepal
UKAID: United Kingdom Agency for International Development
WHO: World Health Organization
WHODAS: World Health Organization Disability Assessment Scale
